# (3*E*,5*E*)-3,5-Bis(2-chloro­benzyl­idene)-1-propyl­piperidin-4-one

**DOI:** 10.1107/S1600536812049252

**Published:** 2012-12-08

**Authors:** Quanzhi Yang, Lingzi Chen, Bixia Weng, Lei Fan, Xiaoping Wu

**Affiliations:** a School of Pharmacy, Wenzhou Medical College, Wenzhou, Zhejiang Province 325035, People’s Republic of China

## Abstract

The title compound, C_22_H_21_Cl_2_NO, is a derivative of mono-carbonyl analogues of curcumin (MACs). The mol­ecule has an *E* conformation for each of the olefinic bonds. The 1-propyl­piperidin-4-one ring has a distorted chair conformation with the ring N and the C and O atoms of the carbonyl group deviating from the mean plane of the remaining four ring C atoms by 0.682 (2), −0.134 (3) and −0.340 (4) Å, respectively. The dihedral angle between the benzene rings is 26.5 (1)°. In the crystal, mol­ecules are connected by weak C—H⋯O and C—H⋯π inter­actions.

## Related literature

For related structures, see: Agrawal & Mishra (2010 ▶); Liang *et al.* (2008 ▶, 2009 ▶); Wu *et al.* (2010 ▶, 2011 ▶); Zhao *et al.* (2010 ▶, 2012 ▶). For background to and applications of chalcones, see: Agrawal & Mishra (2010 ▶); Wu *et al.* (2010 ▶, 2011 ▶); Zhao *et al.* (2012 ▶).
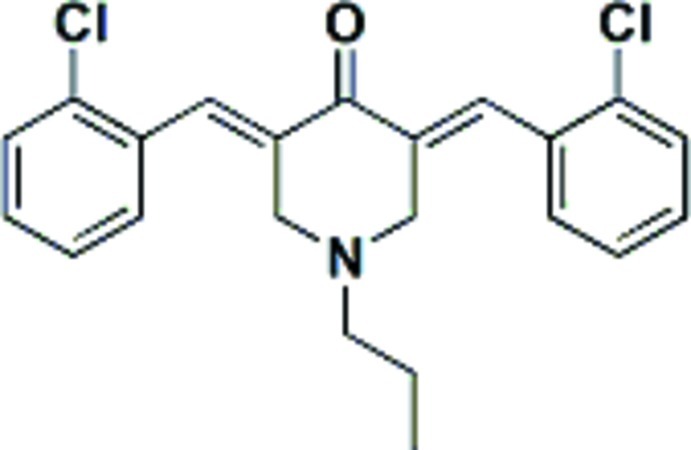


## Experimental

### 

#### Crystal data

C_22_H_21_Cl_2_NO
*M* *_r_* = 386.30
Orthorhombic, 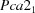

*a* = 18.0123 (15) Å
*b* = 7.0128 (6) Å
*c* = 15.4364 (13) Å
*V* = 1949.9 (3) Å^3^
*Z* = 4
Mo *K*α radiation
μ = 0.34 mm^−1^
*T* = 293 K
0.29 × 0.21 × 0.11 mm

#### Data collection

Bruker SMART CCD area-detector diffractometer
Absorption correction: multi-scan (*SADABS*; Bruker, 2002 ▶) *T*_min_ = 0.759, *T*_max_ = 1.000
11133 measured reflections
3807 independent reflections
3508 reflections with *I* > 2σ(*I*)
*R*_int_ = 0.031

#### Refinement

*R*[*F* ^2^ > 2σ(*F* ^2^)] = 0.035
*wR*(*F* ^2^) = 0.088
*S* = 1.04
3807 reflections
236 parameters
1 restraint
H-atom parameters constrained
Δρ_max_ = 0.22 e Å^−3^
Δρ_min_ = −0.14 e Å^−3^
Absolute structure: Flack (1983 ▶), 1812 Friedel pairs
Flack parameter: 0.01 (5)

### 

Data collection: *SMART* (Bruker, 2002 ▶); cell refinement: *SAINT* (Bruker, 2002 ▶); data reduction: *SHELXTL* (Sheldrick, 2008 ▶); program(s) used to solve structure: *SHELXS97* (Sheldrick, 2008 ▶); program(s) used to refine structure: *SHELXL97* (Sheldrick, 2008 ▶); molecular graphics: *SHELXTL*; software used to prepare material for publication: *SHELXTL*.

## Supplementary Material

Crystal structure: contains datablock(s) I, global. DOI: 10.1107/S1600536812049252/zq2188sup1.cif

Structure factors: contains datablock(s) I. DOI: 10.1107/S1600536812049252/zq2188Isup2.hkl

Supplementary material file. DOI: 10.1107/S1600536812049252/zq2188Isup3.cml

Additional supplementary materials:  crystallographic information; 3D view; checkCIF report

## Figures and Tables

**Table 1:** Hydrogen-bond geometry (Å, °) *Cg*1 is the centroid of the C14–C19 ring.

*D*—H⋯*A*	*D*—H	H⋯*A*	*D*⋯*A*	*D*—H⋯*A*
C20—H20*A*⋯O1^i^	0.97	2.64	3.607 (3)	174
C8—H8⋯*Cg*1^ii^	0.93	2.89	3.568	131
C21—H21*B*⋯*Cg*1^iii^	0.97	3.01	3.613	121

Symmetry codes: (i) 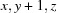; (ii) 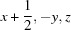; (iii) 
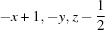
.

**Fig. 1.**
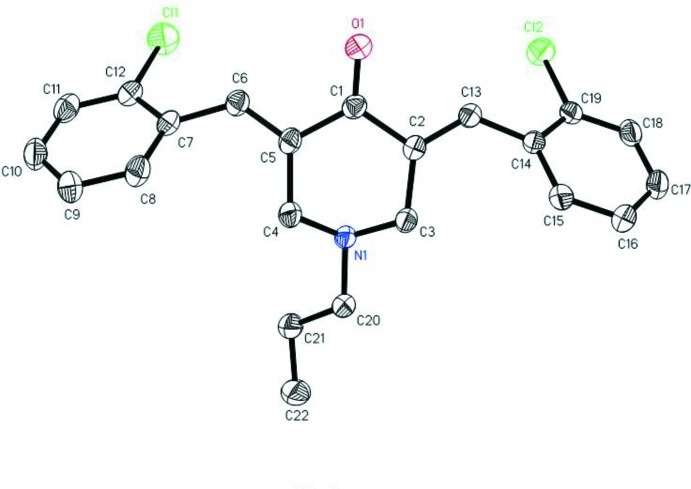
A view of the title complex, showing the atom-numbering scheme and 50% probability displacement ellipsoids (H atoms omitted for clarity).

**Fig. 2.**
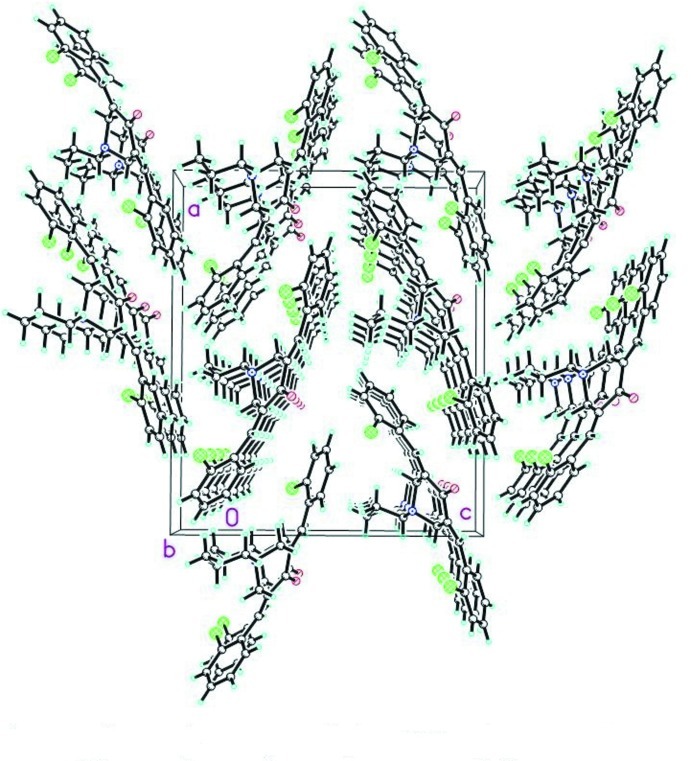
Packing diagram of the title compound along the b axis.

## supplementary crystallographic information

### Comment

Curcumin is a natural drug isolated from turmeric. Curcumin possesses extensive biological activity such as anti-inflammatory, antioxidant, antitumor (Agrawal & Mishra, 2010; Zhao *et al.*, 2012). However, its β-diketone moiety causes instability and poor bioavailability in body. Structural modification to prepare the analogues without the β-diketone moiety leads to mono-carbonyl analogues of curcumin (MACs) which are more stable and possess good pharmacokinetic profiles (Zhao *et al.*, 2012). We synthesized a series of MACs in order to study antitumor and anti-inflammatory activities. In previous researches of our group, we reported the crystal structures of series of 5-carbon-linker mono-carbonyl analogues as following: 1,5-diaryl-1,4-pentadiene-3-ones, 2,6-(diarylidene)cyclohexanone, 2,5-(diarylidene)cyclopentanone (Liang *et al.*, 2008, 2009; Wu *et al.*, 2010, 2011; Zhao *et al.*, 2010, 2012).

In this paper, we present the crystal structure of the title curcumin derivative, C_22_H_21_Cl_2_NO. The molecule has an *E* conformation for each of the olefinic bonds (Fig. 1). The 1-propylpiperidin-4-one ring has a distorted chair conformation: atoms N1, C1 and O1 deviate from the mean plane (C2-C3-C4-C5) by +0.682 (2), -0.134 (3) and -0.340 (4) Å, respectively. The dihedral angle between the phenyl rings C7-C12 and C14-C19 is 26.5 (1)°. In the crystal, molecules are connected by weak C—H···O and C–H···π interactions (Table 1).

### Experimental

The title compound was synthesized by aldol condensation between 1-propylpiperidin-4-one and 2-fluorobenzaldehyde. 1-Propylpiperidin-4-one (1 mmol) and 2-fluorobenzaldehyde (2.1 mmol) were dissolved in absolute ethyl alcohol (20 ml). When the temperature of solution was at 288 K by using ice-water bath, 5 drops of NaOH (40%) were added and the reaction solution became yellow. The reaction was monitored by thin-layer chromatography. After the reaction was over, 20 ml H_2_O was added and the yellow solid was separated out. Precipitation was filtered and washed with mixture of cold ethanol and water (1:10). The production was purified by silica gel column chromatograph, elution solvent was the mixture of petroleum ether and ethyl acetate (4:1). The yield of production is 76.6%, and the melting point is in the range 397.35 – 401.05 K. The pure product was crystallized in the solution mixture of CH_2_Cl_2_ and CH_3_CH_2_OH.

### Refinement

All H atoms were positioned geometrically and refined using a riding model approximation, with C—H = 0.93 Å and *U*_iso_(H) = 1.2*U*_eq_(C) for the aromatic H atoms, with C—H = 0.97 Å and *U*_iso_(H) = 1.2*U*_eq_(C) for the methylene H atoms, and with C—H = 0.96 Å and *U*_iso_(H) = 1.5*U*_eq_(C) for the methyl H atoms.

### Crystal data

**Table:**

C_22_H_21_Cl_2_NO	*F*(000) = 808
*M**_r_* = 386.30	*D*_x_ = 1.316 Mg m^−^^3^
Orthorhombic, *P**c**a*2_1_	Mo *K*α radiation, λ = 0.71073 Å
Hall symbol: P 2c -2ac	Cell parameters from 4766 reflections
*a* = 18.0123 (15) Å	θ = 5.2–54.3°
*b* = 7.0128 (6) Å	µ = 0.34 mm^−^^1^
*c* = 15.4364 (13) Å	*T* = 293 K
*V* = 1949.9 (3) Å^3^	Prismatic, green
*Z* = 4	0.29 × 0.21 × 0.11 mm

### Data collection

**Table:**

Bruker SMART CCD area-detector diffractometer	3807 independent reflections
Radiation source: fine-focus sealed tube	3508 reflections with *I* > 2σ(*I*)
Graphite monochromator	*R*_int_ = 0.031
φ and ω scans	θ_max_ = 26.0°, θ_min_ = 2.3°
Absorption correction: multi-scan (*SADABS*; Bruker, 2002)	*h* = −21→22
*T*_min_ = 0.759, *T*_max_ = 1.000	*k* = −6→8
11133 measured reflections	*l* = −19→19

### Refinement

**Table:**

Refinement on *F*^2^	Secondary atom site location: difference Fourier map
Least-squares matrix: full	Hydrogen site location: inferred from neighbouring sites
*R*[*F*^2^ > 2σ(*F*^2^)] = 0.035	H-atom parameters constrained
*wR*(*F*^2^) = 0.088	*w* = 1/[σ^2^(*F*_o_^2^) + (0.0548*P*)^2^ + 0.0691*P*] where *P* = (*F*_o_^2^ + 2*F*_c_^2^)/3
*S* = 1.04	(Δ/σ)_max_ = 0.001
3807 reflections	Δρ_max_ = 0.22 e Å^−^^3^
236 parameters	Δρ_min_ = −0.14 e Å^−^^3^
1 restraint	Absolute structure: Flack (1983), 1812 Friedel pairs
Primary atom site location: structure-invariant direct methods	Flack parameter: 0.01 (5)

### Special details

**Table:**

Geometry. All e.s.d.'s (except the e.s.d. in the dihedral angle between two l.s. planes) are estimated using the full covariance matrix. The cell e.s.d.'s are taken into account individually in the estimation of e.s.d.'s in distances, angles and torsion angles; correlations between e.s.d.'s in cell parameters are only used when they are defined by crystal symmetry. An approximate (isotropic) treatment of cell e.s.d.'s is used for estimating e.s.d.'s involving l.s. planes.
Refinement. Refinement of *F*^2^ against ALL reflections. The weighted *R*-factor *wR* and goodness of fit *S* are based on *F*^2^, conventional *R*-factors *R* are based on *F*, with *F* set to zero for negative *F*^2^. The threshold expression of *F*^2^ > σ(*F*^2^) is used only for calculating *R*-factors(gt) *etc*. and is not relevant to the choice of reflections for refinement. *R*-factors based on *F*^2^ are statistically about twice as large as those based on *F*, and *R*- factors based on ALL data will be even larger.

### Fractional atomic coordinates and isotropic or equivalent isotropic displacement parameters (Å^2^)

**Table:**

	*x*	*y*	*z*	*U*_iso_*/*U*_eq_	
Cl1	0.78182 (4)	−0.45470 (9)	0.63636 (5)	0.0796 (2)	
Cl2	0.37574 (3)	−0.55096 (7)	0.88228 (4)	0.06296 (16)	
N1	0.56671 (8)	0.1169 (2)	0.77359 (10)	0.0401 (3)	
O1	0.63228 (8)	−0.3652 (2)	0.89850 (10)	0.0607 (4)	
C1	0.61494 (10)	−0.2139 (3)	0.86622 (12)	0.0429 (4)	
C2	0.53972 (9)	−0.1287 (2)	0.88170 (12)	0.0403 (4)	
C3	0.52682 (10)	0.0724 (3)	0.85301 (11)	0.0437 (4)	
H3A	0.4741	0.0920	0.8438	0.052*	
H3B	0.5427	0.1588	0.8984	0.052*	
C4	0.64621 (10)	0.0972 (3)	0.78692 (14)	0.0474 (4)	
H4A	0.6619	0.1817	0.8332	0.057*	
H4B	0.6722	0.1343	0.7345	0.057*	
C5	0.66643 (9)	−0.1048 (3)	0.80985 (12)	0.0423 (4)	
C6	0.72557 (10)	−0.1989 (3)	0.78005 (13)	0.0449 (4)	
H6	0.7282	−0.3274	0.7945	0.054*	
C7	0.78713 (10)	−0.1247 (3)	0.72710 (11)	0.0426 (4)	
C8	0.81962 (12)	0.0505 (3)	0.74401 (14)	0.0541 (5)	
H8	0.7998	0.1274	0.7873	0.065*	
C9	0.88044 (12)	0.1138 (4)	0.69847 (16)	0.0621 (6)	
H9	0.9013	0.2317	0.7114	0.075*	
C10	0.91043 (12)	0.0031 (4)	0.63378 (17)	0.0639 (6)	
H10	0.9516	0.0458	0.6031	0.077*	
C11	0.87924 (12)	−0.1711 (4)	0.61467 (15)	0.0585 (5)	
H11	0.8987	−0.2461	0.5705	0.070*	
C12	0.81917 (11)	−0.2330 (3)	0.66148 (12)	0.0477 (4)	
C13	0.48853 (10)	−0.2435 (3)	0.91708 (11)	0.0416 (4)	
H13	0.5036	−0.3681	0.9278	0.050*	
C14	0.41197 (10)	−0.1981 (3)	0.94103 (11)	0.0407 (4)	
C15	0.39186 (13)	−0.0247 (3)	0.97859 (13)	0.0533 (5)	
H15	0.4275	0.0699	0.9857	0.064*	
C16	0.31982 (14)	0.0090 (4)	1.00541 (16)	0.0658 (6)	
H16	0.3074	0.1254	1.0303	0.079*	
C17	0.26585 (13)	−0.1308 (4)	0.99520 (16)	0.0650 (6)	
H17	0.2174	−0.1081	1.0134	0.078*	
C18	0.28397 (11)	−0.3015 (3)	0.95846 (14)	0.0548 (5)	
H18	0.2481	−0.3956	0.9516	0.066*	
C19	0.35581 (10)	−0.3336 (3)	0.93162 (12)	0.0443 (4)	
C20	0.54687 (11)	0.3110 (3)	0.74676 (12)	0.0460 (4)	
H20A	0.5660	0.4001	0.7893	0.055*	
H20B	0.4932	0.3224	0.7465	0.055*	
C21	0.57591 (14)	0.3668 (3)	0.65862 (15)	0.0645 (6)	
H21A	0.6297	0.3709	0.6603	0.077*	
H21B	0.5614	0.2709	0.6166	0.077*	
C22	0.54679 (16)	0.5583 (3)	0.63016 (19)	0.0746 (7)	
H22A	0.4937	0.5525	0.6249	0.112*	
H22B	0.5681	0.5912	0.5751	0.112*	
H22C	0.5600	0.6531	0.6723	0.112*	

### Atomic displacement parameters (Å^2^)

**Table:**

	*U*^11^	*U*^22^	*U*^33^	*U*^12^	*U*^13^	*U*^23^
Cl1	0.0878 (4)	0.0597 (4)	0.0914 (4)	−0.0037 (3)	0.0140 (4)	−0.0267 (3)
Cl2	0.0552 (3)	0.0512 (3)	0.0825 (4)	−0.0052 (2)	0.0007 (3)	−0.0101 (3)
N1	0.0345 (7)	0.0385 (7)	0.0474 (7)	−0.0002 (6)	0.0026 (6)	−0.0018 (6)
O1	0.0486 (8)	0.0557 (8)	0.0777 (10)	0.0103 (6)	0.0083 (7)	0.0159 (8)
C1	0.0367 (9)	0.0427 (9)	0.0494 (10)	0.0020 (7)	−0.0036 (7)	−0.0021 (8)
C2	0.0364 (8)	0.0445 (9)	0.0399 (8)	0.0012 (7)	0.0007 (7)	−0.0042 (8)
C3	0.0384 (9)	0.0438 (9)	0.0489 (10)	0.0014 (7)	0.0073 (7)	−0.0042 (7)
C4	0.0351 (9)	0.0475 (10)	0.0596 (11)	−0.0032 (8)	0.0020 (8)	−0.0013 (9)
C5	0.0311 (9)	0.0487 (10)	0.0472 (9)	−0.0006 (7)	−0.0034 (7)	−0.0050 (8)
C6	0.0365 (9)	0.0485 (10)	0.0496 (9)	0.0005 (7)	−0.0017 (8)	−0.0029 (8)
C7	0.0339 (9)	0.0489 (10)	0.0451 (9)	0.0068 (7)	−0.0036 (7)	−0.0020 (8)
C8	0.0389 (11)	0.0635 (13)	0.0600 (12)	−0.0005 (9)	−0.0006 (9)	−0.0133 (10)
C9	0.0427 (11)	0.0660 (13)	0.0776 (15)	−0.0089 (10)	0.0006 (10)	0.0011 (12)
C10	0.0419 (11)	0.0811 (15)	0.0688 (13)	−0.0005 (10)	0.0107 (10)	0.0121 (12)
C11	0.0520 (12)	0.0744 (15)	0.0490 (10)	0.0166 (10)	0.0083 (9)	−0.0003 (10)
C12	0.0417 (10)	0.0518 (11)	0.0495 (10)	0.0078 (8)	−0.0044 (8)	−0.0035 (8)
C13	0.0389 (9)	0.0433 (9)	0.0425 (9)	0.0027 (7)	0.0016 (7)	−0.0009 (7)
C14	0.0400 (9)	0.0430 (9)	0.0391 (8)	0.0054 (7)	0.0028 (7)	0.0062 (7)
C15	0.0591 (12)	0.0473 (11)	0.0535 (11)	0.0017 (9)	0.0146 (10)	0.0030 (9)
C16	0.0690 (15)	0.0582 (12)	0.0703 (14)	0.0132 (11)	0.0272 (12)	0.0026 (11)
C17	0.0487 (12)	0.0756 (15)	0.0708 (14)	0.0113 (11)	0.0225 (11)	0.0160 (12)
C18	0.0394 (10)	0.0651 (13)	0.0598 (11)	−0.0013 (9)	0.0070 (8)	0.0163 (10)
C19	0.0431 (9)	0.0449 (10)	0.0450 (9)	0.0029 (8)	0.0027 (8)	0.0097 (8)
C20	0.0436 (10)	0.0434 (10)	0.0508 (10)	0.0028 (8)	0.0052 (8)	−0.0024 (8)
C21	0.0701 (15)	0.0563 (13)	0.0671 (13)	0.0111 (10)	0.0247 (12)	0.0092 (10)
C22	0.0879 (18)	0.0614 (14)	0.0746 (15)	0.0123 (11)	0.0157 (14)	0.0184 (12)

### Geometric parameters (Å, º)

**Table:**

Cl1—C12	1.738 (2)	C10—H10	0.9300
Cl2—C19	1.741 (2)	C11—C12	1.372 (3)
N1—C4	1.453 (2)	C11—H11	0.9300
N1—C3	1.455 (2)	C13—C14	1.463 (2)
N1—C20	1.466 (2)	C13—H13	0.9300
O1—C1	1.213 (2)	C14—C15	1.395 (3)
C1—C5	1.484 (3)	C14—C19	1.396 (3)
C1—C2	1.500 (2)	C15—C16	1.382 (3)
C2—C13	1.340 (2)	C15—H15	0.9300
C2—C3	1.497 (3)	C16—C17	1.390 (4)
C3—H3A	0.9700	C16—H16	0.9300
C3—H3B	0.9700	C17—C18	1.364 (3)
C4—C5	1.505 (3)	C17—H17	0.9300
C4—H4A	0.9700	C18—C19	1.377 (3)
C4—H4B	0.9700	C18—H18	0.9300
C5—C6	1.335 (3)	C20—C21	1.509 (3)
C6—C7	1.472 (3)	C20—H20A	0.9700
C6—H6	0.9300	C20—H20B	0.9700
C7—C8	1.386 (3)	C21—C22	1.507 (3)
C7—C12	1.391 (3)	C21—H21A	0.9700
C8—C9	1.375 (3)	C21—H21B	0.9700
C8—H8	0.9300	C22—H22A	0.9600
C9—C10	1.376 (4)	C22—H22B	0.9600
C9—H9	0.9300	C22—H22C	0.9600
C10—C11	1.377 (3)		
			
C4—N1—C3	110.30 (15)	C11—C12—C7	122.6 (2)
C4—N1—C20	111.63 (14)	C11—C12—Cl1	118.09 (16)
C3—N1—C20	108.46 (14)	C7—C12—Cl1	119.35 (16)
O1—C1—C5	122.06 (16)	C2—C13—C14	128.35 (17)
O1—C1—C2	121.03 (17)	C2—C13—H13	115.8
C5—C1—C2	116.91 (15)	C14—C13—H13	115.8
C13—C2—C3	125.45 (16)	C15—C14—C19	116.66 (17)
C13—C2—C1	116.55 (15)	C15—C14—C13	122.67 (18)
C3—C2—C1	117.94 (15)	C19—C14—C13	120.57 (17)
N1—C3—C2	112.02 (14)	C16—C15—C14	121.1 (2)
N1—C3—H3A	109.2	C16—C15—H15	119.4
C2—C3—H3A	109.2	C14—C15—H15	119.4
N1—C3—H3B	109.2	C15—C16—C17	120.2 (2)
C2—C3—H3B	109.2	C15—C16—H16	119.9
H3A—C3—H3B	107.9	C17—C16—H16	119.9
N1—C4—C5	111.19 (15)	C18—C17—C16	119.9 (2)
N1—C4—H4A	109.4	C18—C17—H17	120.0
C5—C4—H4A	109.4	C16—C17—H17	120.0
N1—C4—H4B	109.4	C17—C18—C19	119.6 (2)
C5—C4—H4B	109.4	C17—C18—H18	120.2
H4A—C4—H4B	108.0	C19—C18—H18	120.2
C6—C5—C1	116.51 (18)	C18—C19—C14	122.58 (18)
C6—C5—C4	125.24 (17)	C18—C19—Cl2	117.92 (16)
C1—C5—C4	118.16 (15)	C14—C19—Cl2	119.49 (14)
C5—C6—C7	128.18 (18)	N1—C20—C21	114.26 (16)
C5—C6—H6	115.9	N1—C20—H20A	108.7
C7—C6—H6	115.9	C21—C20—H20A	108.7
C8—C7—C12	116.48 (18)	N1—C20—H20B	108.7
C8—C7—C6	121.77 (17)	C21—C20—H20B	108.7
C12—C7—C6	121.59 (18)	H20A—C20—H20B	107.6
C9—C8—C7	121.8 (2)	C22—C21—C20	111.92 (18)
C9—C8—H8	119.1	C22—C21—H21A	109.2
C7—C8—H8	119.1	C20—C21—H21A	109.2
C8—C9—C10	120.1 (2)	C22—C21—H21B	109.2
C8—C9—H9	119.9	C20—C21—H21B	109.2
C10—C9—H9	119.9	H21A—C21—H21B	107.9
C9—C10—C11	119.8 (2)	C21—C22—H22A	109.5
C9—C10—H10	120.1	C21—C22—H22B	109.5
C11—C10—H10	120.1	H22A—C22—H22B	109.5
C12—C11—C10	119.3 (2)	C21—C22—H22C	109.5
C12—C11—H11	120.3	H22A—C22—H22C	109.5
C10—C11—H11	120.3	H22B—C22—H22C	109.5
			
O1—C1—C2—C13	−13.3 (3)	C10—C11—C12—C7	1.3 (3)
C5—C1—C2—C13	166.64 (16)	C10—C11—C12—Cl1	−179.34 (18)
O1—C1—C2—C3	169.50 (17)	C8—C7—C12—C11	−0.7 (3)
C5—C1—C2—C3	−10.6 (2)	C6—C7—C12—C11	−176.22 (19)
C4—N1—C3—C2	−61.51 (19)	C8—C7—C12—Cl1	179.91 (15)
C20—N1—C3—C2	175.95 (15)	C6—C7—C12—Cl1	4.4 (2)
C13—C2—C3—N1	−141.92 (17)	C3—C2—C13—C14	−4.6 (3)
C1—C2—C3—N1	35.0 (2)	C1—C2—C13—C14	178.42 (17)
C3—N1—C4—C5	62.3 (2)	C2—C13—C14—C15	−39.3 (3)
C20—N1—C4—C5	−177.01 (15)	C2—C13—C14—C19	144.50 (18)
O1—C1—C5—C6	14.9 (3)	C19—C14—C15—C16	0.4 (3)
C2—C1—C5—C6	−164.98 (16)	C13—C14—C15—C16	−175.87 (19)
O1—C1—C5—C4	−168.33 (18)	C14—C15—C16—C17	0.0 (3)
C2—C1—C5—C4	11.8 (2)	C15—C16—C17—C18	−0.1 (4)
N1—C4—C5—C6	139.18 (19)	C16—C17—C18—C19	−0.1 (3)
N1—C4—C5—C1	−37.3 (2)	C17—C18—C19—C14	0.5 (3)
C1—C5—C6—C7	−176.63 (18)	C17—C18—C19—Cl2	−178.21 (17)
C4—C5—C6—C7	6.9 (3)	C15—C14—C19—C18	−0.7 (3)
C5—C6—C7—C8	42.7 (3)	C13—C14—C19—C18	175.70 (17)
C5—C6—C7—C12	−142.0 (2)	C15—C14—C19—Cl2	178.04 (14)
C12—C7—C8—C9	−0.2 (3)	C13—C14—C19—Cl2	−5.6 (2)
C6—C7—C8—C9	175.4 (2)	C4—N1—C20—C21	66.3 (2)
C7—C8—C9—C10	0.4 (4)	C3—N1—C20—C21	−171.97 (18)
C8—C9—C10—C11	0.1 (4)	N1—C20—C21—C22	173.6 (2)
C9—C10—C11—C12	−1.0 (4)		

### Hydrogen-bond geometry (Å, º)

Cg1 is the centroid of the C14–C19 ring.

**Table:**

*D*—H···*A*	*D*—H	H···*A*	*D*···*A*	*D*—H···*A*
C20—H20*A*···O1^i^	0.97	2.64	3.607 (3)	174
C8—H8···*Cg*1^ii^	0.93	2.89	3.568	131
C21—H21*B*···*Cg*1^iii^	0.97	3.01	3.613	121

Symmetry codes: (i) *x*, *y*+1, *z*; (ii) *x*+1/2, −*y*, *z*; (iii) −*x*+1, −*y*, *z*−1/2.

## References

[bb1] Agrawal, D. K. & Mishra, P. K. (2010). *Med. Res. Rev.* **30**, 818–860. 10.1002/med.20188 20027668

[bb2] Bruker (2002). *SMART*, *SAINT* and *SADABS* Bruker AXS Inc., Madison, Wisconsin, USA.

[bb3] Flack, H. D. (1983). *Acta Cryst.* A**39**, 876–881.

[bb4] Liang, G., Yang, S. L., Shao, L. L., Zhao, C. G., Xiao, J., Lv, Y. X., Yang, J., Zhao, Y. & Li, X. K. (2008). *J. Asian Nat. Prod. Res.* **10**, 957–965. 10.1080/10286020802181257 19003615

[bb5] Liang, G., Yang, S., Zhou, H., Shao, L., Huang, K., Xiao, J., Huang, Z. & Li, X. (2009). *Eur. J. Med. Chem.* **44**, 915–919. 10.1016/j.ejmech.2008.01.031 18336957

[bb6] Sheldrick, G. M. (2008). *Acta Cryst.* A**64**, 112–122. 10.1107/S0108767307043930 18156677

[bb7] Wu, J., Li, J., Cai, Y., Pan, Y., Ye, F., Zhang, Y., Zhao, Y., Yang, S., Li, X. & Liang, G. (2011). *J. Med. Chem.* **54**, 8110–8123. 10.1021/jm200946h 21988173

[bb8] Wu, J., Wang, C., Cai, Y., Yang, S., Zheng, X., Qiu, P., Peng, J., Wu, X., Liang, G. & Li, X. (2010). *Chin. J. Org. Chem.* **30**, 884–889.

[bb9] Zhao, C., Cai, Y., He, X., Li, J., Zhang, L., Wu, J., Zhao, Y., Yang, S., Li, X., Li, W. & Liang, G. (2010). *Eur. J. Med. Chem.* **45**, 5773–5780. 10.1016/j.ejmech.2010.09.037 20934787

[bb10] Zhao, C., Liu, Z. & Liang, G. (2012). *Curr. Pharm. Des.* In the press.

